# Rasch validation of the short form (8 item) PC-QoL questionnaire and applicability of use as a health state classification system for a new preference-based measure

**DOI:** 10.1007/s11136-024-03652-w

**Published:** 2024-04-23

**Authors:** Jack M. Roberts, Anne B. Chang, Vikas Goyal, Nitin Kapur, Julie M. Marchant, Steven M. McPhail, Sanjeewa Kularatna

**Affiliations:** 1 Australian Centre for Health Services Innovation, Centre for Healthcare Transformation, School of Public Health and Social Work, Queensland University of Technology, Brisbane, Australia; 2 NHMRC Centre for Research Excellence in Paediatric Bronchiectasis (AusBREATHE), Menzies School of Health Research, Darwin, Australia; 3 Department of Respiratory and Sleep Medicine, Queensland Children’s Hospital, Brisbane, Australia; 4 Digital Health and Informatics Directorate, Metro South Health, Brisbane, Australia; 5 Health Services and Systems Research, Duke-NUS Medical School, Singapore, Singapore

**Keywords:** Chronic cough, Quality of life, Paediatrics, Patient-reported outcomes, Health utility, Quality-adjusted life years

## Abstract

**Background:**

The parent-proxy paediatric chronic cough quality of life questionnaire (PC-QoL) is a commonly used measure of spillover quality of life in parents of children with chronic cough. To date, spillover health utility in these parents is not routinely estimated largely due to the lack of a suitable instrument. Their perspective is not included in economic evaluations of interventions for their children. We explored developing a health state classification system based on the PC-QoL for measuring health utility spill over in this population.

**Methods:**

This study included PC-QoL 8-item responses of 653 parents participating in a prospective cohort study about paediatric chronic cough. Exploratory factor analysis (EFA) and Rasch analysis were used to examine dimensionality and select potential items and level structure.

**Results:**

EFA indicated that the PC-QoL had one underlying domain. Rasch analysis indicated threshold disordering in all items which improved when items were collapsed from seven to four levels. Two demonstrated differential item functioning (DIF) by diagnosis or ethnicity and were excluded from the final scale. This scale satisfied Rasch assumptions of local independence and unidimensionality and demonstrated acceptable fit to the Rasch model. It was presented to and modified by an expert panel and a consumer panel. The resulting classification system had six items, each with four levels.

**Discussion:**

The PC-QoL can conform to a Rasch model with minor modifications. It may be a good basis for the classification system of a child cough-specific PBM. A valuation study is required to estimate preference weights for each item and to estimate health utility in parents of children with chronic cough.

**Supplementary Information:**

The online version contains supplementary material available at 10.1007/s11136-024-03652-w.

## Plain English summary

Chronic cough (lasting more than four weeks) in children is an important issue in healthcare because it may be a sign of underlying disease and is a common reason for parents to seek medical assistance. Evidence-based guidelines advocate assessment and early appropriate treatment as it leads to improved quality of life in children and their parents and may prevent further lung damage. However, there is not much data on the economic benefits of treatment that include measured quality-adjusted life years, as there is an absence of any instrument suitable to do this that is specific for children with chronic cough.

In this study, we examined a commonly used questionnaire that measures the quality of life of parents of children with chronic cough called the paediatric chronic cough quality of life questionnaire (PC-QoL) to see if it could be used in a format from which quality-adjusted life years could be estimated. Using statistical analyses, we found that some questions from the PC-QoL have potential to be used to estimate the quality-adjusted life years in children with chronic cough. An important next step for estimating quality-adjusted life years in children with chronic cough will be to undertake further research to estimate how the many possible health states that can be described for children with chronic cough are valued by society.

## Background

Chronic cough in children, which has been defined as a cough lasting for longer than four weeks, is a common reason for presentation to healthcare professionals [1, 2]. Chronic cough may indicate underlying lung disease, e.g. protracted bacterial bronchitis (PBB), cystic fibrosis, bronchiectasis, chronic suppurative lung disease (CSLD) and interstitial lung disease [3]. The symptom of chronic cough is associated with high treatment and economic burden. It can also lead to the detriment of health-related quality of life (HR-QoL) in both children and their families [2, 4]. Additionally, some chronic conditions associated with non-optimally managed chronic cough, including bronchiectasis and CSLD, may also be associated with high costs and poor HR-QoL [2, 5–7], may be avoided with successful early intervention [8–10].

HR-QoL measures are patient-reported outcome measures that capture a patient’s (or their family’s) quality of life at a given point in time. HR-QoL measures capture physical, emotional and social function from the perspective of the respondent based on their own lived experiences in a way that may be otherwise overlooked by health professionals [11–13]. HR-QoL is also a core outcome for research studies of chronic cough and bronchiectasis [14, 15].

HR-QoL measures can be used to quantify health states in ways that allow the lived experiences of a person to be included in economic evaluations, through the use of quality-adjusted life years (also known as QALYs). However, this requires additional development steps and research that allows for the assignment of societal preference weights for each possible combination of item responses from an instrument. Instruments that have had these adaptations and societal preference values are known as preference-based measures [16]. The summary score of a preference-based measure is health utility, commonly anchored by the health states of full health (represented by 1.00) and dead (represented by 0.00). QALYs can be estimated by multiplying health utility derived from a preference-based measure by the duration of time in years lived in that health state [17]. Additionally, there has been interest the inclusion in economic analyses of the effect a condition may have on carers or other people who are close to the patient, termed spill over effect [18]. QALYs are the measure of health state benefit in cost-utility analyses, which are a special case of cost-effectiveness, widely used for informing healthcare policy and resource allocation decisions.

HR-QoL measures and preference-based measures are broadly categorised as either generic or specific (either for a condition/disease and/or a population). Generic instruments are thought to be comparable between conditions, simple to administer and the psychometric properties of many instruments including the preference-based EQ-5D-Y and the CHU-9D have been validated in children [19, 20]. However, generic quality of life instruments have received criticism for lacking sensitivity, particularly in relation to important outcomes specific to particular conditions [21], whereas condition or disease-specific quality of life instruments are potentially more sensitive in detecting differences in quality of life amongst people with the condition of interest [21, 22]. Additionally, being able to derive both condition-specific health-related quality of life estimates and health utility estimates from a single condition-specific instruments has the potential to reduce respondent burden in comparison to completing additional generic preference-based measures for the purpose of estimating QALYs. For these reasons, the adaptation of widely used condition-specific patient-reported outcomes for use as preference-based measure has become increasingly common [23–26].

The parent-proxy, chronic cough-specific quality of life questionnaire (PC-QoL) is a specific HR-QoL questionnaire for parents of children with chronic cough [27]. Whilst it is named a proxy measure, it may be more accurately described as a measure of the spillover effects of cough on the QoL of parents [28]. Evidence supports the PC-QoL’s reliability, including high internal consistency, test–retest intraclass correlation and test–retest reliability [27, 29] and validity, including convergent validity with cough frequency/severity measures, the DASS-21, 5 domains of SF-12 and PedsQL [27, 29]. Additionally, the PC-QoL is responsive to changes in the parent’s HR-QoL as the child’s cough is treated, indicating discriminant validity and sensitivity in this domain e. It has been recommended for use in studies of paediatric chronic cough by a CHEST Expert Panel [15, 30]. The PC-QoL has also been identified as an important measure of a core outcome (HR-QoL) for studies of interventions in protracted bacterial bronchitis [31] and has been used in measuring HR-QoL in children diagnosed with bronchiectasis and chronic suppurative lung disease [32]. It currently exists as a 27-item “long” form and an 8-item “short” form [27–29, 33]. High correlations between the proxy PC-QoL scores and self-reported child chronic cough QoL [34] scores have also been described [5]. The short PC-QoL-8 consists of 8 items (Table 1) regarding the frequency of symptoms and feelings scored on a 7-level Likert scale ranging from “All of the time” (1) to “None of the time” (7), across three domains, social, emotional and physical functioning [27]. It is scored by taking the mean of all items with higher scores indicate higher quality of life [27].

**Table 1:** PC-QoL-8 item [34]

Item	Item description (“During the past week how often…”)
Item 1	Did you feel **helpless** because of your child’s cough?
Item 2	Were you worried/concerned about your **child not sleeping** because of cough?
Item 3	Did you feel **overprotective** because of your child’s cough?
Item 4	Did you feel **upset** because of your child’s cough?
Item 5	Were you worried/concerned about **leaving your child with others** because of their cough?
Item 6	Did you feel **scared** because of your child’s cough?
Item 7	Were you worried/concerned about your child being able to **lead a normal life**?
Item 8	Were you **awakened during the night** because of your child’s cough?

Bold signifies main theme of the item

The PC-QoL has been used in a variety of observational and interventional studies [2, 3, 5, 35, 36] but no value set for a preference-based measure has been derived from PC-QoL items. Having a preference value set for health states described by the PC-QoL could facilitate economic analyses of prior and future studies using this questionnaire. The long and short form of the PC-QoL may form 7^27^ or 7^8^ possible health states, respectively, which would be impractical to value with currently available methods. To address this, our study’s aims were to (a) further explore the classical test theory psychometric properties and the underlying dimensional structure of the PC-QoL-8, (b) explore item and level reduction of the PC-QoL-8 using a Rasch analytical approach, and (c) explore local dependency and dimensional structure of the classification system with a reduced number of items to assist in the avoidance of implausible health states in the valuation study. It is important to note that the number of levels (and therefore possible health states) in rating scales can be artificially reduced by merging neighbouring levels, irrespective of the Rasch model used. These steps are necessary to make the valuation study required for eliciting preference weights feasible.

## Methods

### Participants and setting

We performed a secondary analysis from a subgroup of participants with chronic cough from a prospective cohort study examining children undergoing a flexible bronchoscopy at three hospitals in Brisbane and Darwin in Australia. The methods for the overarching cohort study have been described in detail elsewhere [37, 38]. Briefly, parents and carers of children undergoing flexible bronchoscopy between April 2011 and January 2022 were invited to participate if their child was undergoing bronchoscopy, did not already have an underlying respiratory diagnosis, excluding asthma. We included parents of children aged < 18 years who agreed to participate and had either, “chronic cough” noted as an indication for the bronchoscopy, a parent/carer reported history of ≥ 4 weeks of cough, or who went on to be diagnosed with PBB, bronchiectasis or CSLD. Some participants without a history of chronic cough were included in other analyses of these data but were excluded from the present study [37, 38]. Data were collected using paper forms on the day of bronchoscopy and entered in to either a REDCap database [39] or pre 2020, an SPSS database [40]. The study received ethical approval from the Children’s Health Queensland Human Research Ethics Committee (HREC/03/QRCH/17).

### Materials

Parents and carers were asked to complete a paper version of the PC-QoL-8 questionnaire on the day of bronchoscopy. The 8-item PC-QoL was chosen for this study as it was the most widely used version, and each of the eight items are also present in the long 27-item version. A case report form with questions about the child’s socio-demographic background, cough and medical history and results of the bronchoscopy and concurrent investigations was also completed.

### Analysis

The analysis plan was designed to (a) assess the psychometric properties of the PC-QoL-8 and (b) assess suitability of questionnaire items for future valuation as a preference-based measure. This analysis approach was consistent with the process for developing condition-specific preference-based measure described by Mavranezouli et al. [26].

#### Classical test theory psychometric properties

Demographic, clinical and PC-QoL items were analysed descriptively. A correlation matrix, measures of sampling adequacy table, and Bartlett’s test of Sphericity were calculated for the PC-QoL to assess suitability for factorisation. Factors were then extracted using principal components analysis and the number of factors retained was guided by Kaiser’s Criterion (eigenvalues > 1.0) [41], Catell’s scree test [42] and parallel analysis [43]. Factors were extracted using exploratory factor analysis with minimum residuals, and Cronbach’s alpha was calculated for the scale.

#### Reducing items and levels with a Rasch approach

Rasch analysis was performed using a partial credit model as has been described elsewhere [24, 44]. We followed previously established guidelines for Rasch analysis of a condition-specific instrument for use as a health state classification system of a preference-based measure [26]. Firstly, we explored the effect of item threshold disordering, that is, disordering of the thresholds between category probability curves which describe the probability of a respondent choosing a level given their location on the underlying scale. Threshold disordering may occur in some Rasch models, like the partial credit model used here which we interpreted as an indication that there are too many levels in the item, and respondents were not able to consistently distinguish between adjacent levels or that the difference between adjacent levels is ambiguous. Because one of our goals was to reduce the number of possible health states that the PC-QoL could describe, item threshold disordering was attended to by exploration of the effect of collapsing levels in a clinically feasible way. Goodness of fit of individual items and the scale as a whole model was monitored for deterioration as levels were collapsed to ensure that changes were not detrimental to model fit. When exploring the exclusion of items from the scale we examined whether individual items had fit residuals greater than ± 2.5 or significant *χ*^2^ statistics (0.05 level with Bonferroni adjustment). Items were also considered for exclusion if they demonstrated differential item functioning (whether participant responses depended on child’s age, ethnicity or diagnosis) as has been described in other works [26]. Items with potential differential item functioning were identified statistically by examining the results of ANOVA of residuals in two or more groups in the DIF summary screen in RUMM2030. We graphically examined the item characteristic curves of identified items for evidence of non-uniform differential item functioning (in particular, differing slope of the curves for certain groups).Items meeting the above criteria of differential and scale misfit were excluded iteratively, followed by reanalysis of the fit of the remaining scale to the Rasch model until no more items met the exclusion criteria.

#### Testing local independence and unidimensionality

We tested the assumptions of local independence of the reduced scale (by examination of the residual correlations matrix for correlations greater than 0.3, checking the wording of the item and using the subtest function for each correlated pair to ensure that they did not substantially inflate the person separation index) and unidimensionality (by examination of the residual principal components matrix, items were grouped based on which direction they loaded on to PC1, and the groups were compared using the equating tests/paired *t* tests function).

Conventional psychometric tests were performed using R [45] (using the tidyverse [46], corrplot [47], psych [48], gtsummary [49], lavaan [50] and nFactors packages [51]), and Rasch analysis was performed using RUMM2030 [52].

Finally, the results of this analysis were presented to a panel of experts to explore content validity and discuss the re-admission or removal of any items and the dimensional structure of the questionnaire. The resulting scale was later presented to, and discussed with, a panel of consumers [Australian Bronchiectasis Centre of Research Excellence esp. for Aboriginal and Torres Strait Islander Children Parent and Community Advisory Group (PAG): https://www.crelungs.org.au/cre-parent-and-community-advisory-group] for further input.

## Results

### Participants

Parents of 652 eligible children completed the questionnaire on the day of their child’s bronchoscopy. 371 children were male (57%), and they had a median age of 2.36 (IQR: 1.48, 4.47) years. The most common final respiratory diagnosis after bronchoscopy and other investigations was bronchiectasis (*n* = 324, 50%) (Table 2).

**Table 2:** Demographic and clinical characteristics of children included in sample

Characteristic	*N* = 652
Age—median (IQR)	2.40 (1.48, 4.47)
Sex *n* (%)	
Male	371 (57%)
Female	281 (43%)
First nations status *n* (%)	
Non-Indigenous	462 (71%)
First nations Australian	190 (29%)
Final respiratory diagnosis *n* (%)	
Bronchiectasis	324 (50%)
PBB	218 (33%)
No diagnosis	56 (9%)
CSLD	54 (8%)

### Classical test theory

#### Item acceptability

All items demonstrated high completion rates (low missing rates), indicating that participants likely did not find any item particularly objectionable (Online resource Table S1). All items demonstrated non-normal distribution of responses and two had a high floor (1 = “All of the time”) effect of greater than 18%. All items demonstrated a high ceiling (7 = “None of the time”) effect of greater than 18% (Online resource Table S1, Fig. S1).

#### Internal consistency and factorability

The PC-QoL-8 demonstrated high internal consistency; Cronbach’s alpha was 0.91. Cronbach’s alpha if item dropped was calculated to be lower than 0.91 for each item. We hypothesised that the PC-QoL is unidimensional, measuring HR-QoL in this population. Correlations between all items were greater than 0.4 supporting the hypothesis that each item relates to the underlying QoL construct (Online resource Fig. S2). The correlation between “not sleeping well” and “awakened during the night” are high (0.70), but this was expected given the two concepts are highly related. This was also the case with “scared” and “upset” (Online resource Fig. S2). Mean inter-item correlation was 0.56, mean item total correlation was 0.79, and split half reliability adjusted with spearman-brown prophecy formula was 0.87. Kaiser–Meyer–Olkin overall measure of sampling adequacy was “marvellous” at 0.912 (Online resource Table S2) [53], and Bartlett’s test of sphericity reached statistical significance, indicating suitability of the data for extraction of factors [54]. Lastly, the determinant of the correlation matrix was 0.005 indicating no multicollinearity or singularity was present in the data.

#### Extraction of factors

Confirmatory factor analysis of the scale using the three-factor model proposed by Newcombe et al. [27] was performed and demonstrated poor model fit (Online resource Fig. S3, Table S3). Principal components analysis revealed one underlying component with an eigenvalue greater than one (Online resource Table S4). Parallel analysis of eigenvalues using a randomly generated data matrix of the same size as the present data and Catell’s scree test also supported the extraction of one underlying factor. No approach taken above (scree, Kaiser, or eigenvalue) indicated that this questionnaire required more than one underlying/latent dimension. All items had sufficient loading on to the one factor (Table 3) and 59.6% variance in the scale explained by one factor was also sufficient. We therefore concluded that the scale was unidimensional and thus suitable for Rasch analysis without modification.

**Table 3:** Exploratory factor analysis results of PC-QoL with loading coefficients on to a one-factor model

Item	Factor 1 loading	Variance explained by factor	Residual variance	Hoffman’s index of complexity
Upset	0.807	0.65	0.35	1
Helpless	0.793	0.63	0.37	1
Scared	0.788	0.62	0.38	1
Not sleeping well	0.783	0.61	0.39	1
Overprotective	0.782	0.61	0.39	1
Leaving child with others	0.767	0.59	0.41	1
Lead a normal life	0.733	0.54	0.46	1
Awakened	0.721	0.52	0.48	1

All loading coefficients and variance explained by factor 1 were high supporting the hypothesis of unidimensionality

### Item response theory approach and Rasch analysis

#### Fit statistics of the unmodified scale

We attempted to fit the scale to the Partial Credit Rasch Model. Mean item fit residual of the unmodified scale was 0.520, SD: 1.515 indicating misfit of the items. Mean person fit residual was − 0.508, SD: 1.566 indicating misfit of some persons. Overall fit of the model was not significant (Total item Chi-Square = 75.108, DF = 56, *p* = 0.045060), which was greater than the Bonferroni-adjusted alpha of 0.05/8 = 0.0063 and did not indicate overall item misfit. The person separation statistics PSI was 0.849, acceptable and indicated an ability to statistically differentiate at least two groups of respondents. These statistics indicated some misfit of the model, which were further explored. All items had disordered thresholds. Items 5 (lead a normal life) and 8 (awakened during the night) demonstrated extreme fit residual values of 2.802 and 3.344, respectively, indicating a low level of discrimination. No item demonstrated a chi-square probability below the Bonferroni adjustment with a probability of 0.5.

#### Category thresholds and Response ordering

All items demonstrated category threshold disordering (see Online resource Fig. S4 for category probability curves of unmodified items), which may indicate an inability of participants to distinguish between adjacent levels of each item and could be related to redundancy or ambiguity of levels. This has also been noted in our clinical experience with parents when administering the survey. We explored iteratively collapsing levels to 6, 5 and eventually 4 categories, aiming for a compromise between best model fit and best evenness and ordering of Andrich thresholds. All items were recoded, with items 3, 4, 6, 7 and 3 (overprotective, upset, scared, lead a normal life) recoded from 0123456 to 0011223, item 5 (leaving child with others) recoded to 0112223, and all others recoded to 0112233 and thresholds were ordered on the threshold map (Online resource Fig. S5). Recoding the scale improved item and person mean fit residuals item trait interaction statistics and person separation index were still acceptable and individual item fit residuals were improved (Online resource Table S4).

#### Differential item functioning and item fit residuals

Given that the main goal of this analysis was to reduce the number of possible combinations of items and levels, we decided that any items that demonstrated differential item functioning (DIF) (systemic differences in response patterns by a person factor, such as age, gender, diagnosis or ethnicity) would be considered for exclusion. No DIF was detected for child’s sex, but, item 7 (lead a normal life) demonstrated DIF for age. This item was removed. Re-analysis of the remaining items revealed DIF for indigenous status and diagnosis category for item 1 (helpless), which was also removed. Whilst these modifications improved model fit, they also lowered the person separation index (Table 4). After this modification, item 8 (awakened during the night) still had a high fit residual, and we again tested removal of this item; however, this resulted both in an increase in the person fit residual SD and a decrease in the person separation index, additionally, a 6-item scale with 4 levels in each item could reasonably be amenable to valuation, thus it was decided not to remove item 8 at this time. Thresholds in the reduced scale were still ordered (Figs. S6, S7).

**Table 4:** Results of Rasch analysis after exclusion of items with DIF

Item (“During the past week how often…”)	Statistics after DIF items excluded			
	Residual	*χ*^2^	*p*-value	DIF
2. Were you worried/concerned about your child **not sleeping** because of cough?	0.357	7.982	0.239447	No
3. Did you feel **overprotective** because of your child’s cough?	0.691	10.107	0.120215	No
4. Did you feel **upset** because of your child’s cough?	− 0.724	11.124	0.084607	No
5. Were you worried/concerned about **leaving your child with others** because of their cough?	1.036	4.828	0.566118	No
6. Did you feel **scared** because of your child’s cough?	− 0.588	10.73	0.097076	No
8. Were you **awakened during the night** because of your child’s cough?	2.554^a^	5.668	0.461332	No
Overall model statistics:	Total—item trait *χ*^2^ = 50.440, *p* = 0.056^b^ Person separation index = 0.81 Person location: mean (SD) = 0.107 (1.568) Person fit residual: mean (SD) = − 0.427 (1.340) Item location: mean (SD) = 0.00 (0.273) Item fit residual: mean (SD) = 0.554 (1.202) Person standard error: mean = 0.640729			

Bold signifies main theme of the item

^a^Extreme fit residual value >  ± 2.5 threshold

^b^Bonferroni adjusted alpha: 0.05/6 = 0.00833

^c^Fit residual SD threshold = 1.5

#### Testing assumptions of local independence and unidimensionality

The final step in the Rasch analysis of the PC-QoL was to explore whether the modified scale still met the Rasch assumptions of unidimensionality and local independence. To explore local independence, we consulted the residual correlation matrix. Item 8 (awakened during the night) demonstrated high correlations (> 0.3) with items 3, 5, and 6, (scared, leaving child with others and overprotective), and item 2 (not sleeping well) with item 6 (scared) (Online resource Table S6). We checked the wording of the correlated items and determined this was not the cause of the correlations. A subtest comparing person separation index with and without correlated items demonstrated that the correlations did not substantially inflate person separation index values, we thus concluded that there was no issue of local dependency. To test the assumption of unidimensionality we examined the principal components of item residuals and performed a paired *t* test of items positively correlated with residual principal component positive vs negatively correlated items. 27 cases (4.14%) had statistically significant scores between positive and negatively loaded items, which we considered acceptable, and not indicative of a violation of the assumption of unidimensionality. We also explored item targeting (see Online resource Table Fig. S8, S9).

### Expert panel and consumer discussion

Following the Rasch analysis, the proposed scale was presented to an expert panel of paediatric respiratory physicians (*n* = 4) and health economists with expertise in psychometric measurement and the development of preference-based measurements (*n* = 2). Overall, the amended scale was acceptable to the panel; however, they recommended that “[being] awakened during the night…” be removed, as it likely redundant due to the similar concept of “[being] worried/concerned about [their] child not sleeping well due to their cough…”. They also recommended the re-inclusion of “Worried/concerned about child being able to lead a normal life” citing qualitative research and clinical experience indicating the importance of this concept to parents and children with chronic cough [4, 55]. The wording of the new levels was also discussed, with the panel choosing “always”, “often”, “sometimes” and “never” as the labels for levels 1, 2, 3 and 4, respectively, with increasing levels indicating increasing health-related quality of life (Table 5). The resulting scale was presented to the parent advisory group who felt that whilst “[being] awakened during the night…” and “[being] worried/concerned about [their] child not sleeping well…” were related to a similar sleep related concept, “[being] awakened during the night…” was likely to me more sensitive and relevant to caring for a child with chronic cough and parents cited experiences of themselves being woken by the cough, but not the child. The parent advisory group agreed on the removal of the “helpless” item, citing experience of feelings of helplessness diminishing with diagnosis and treatment. Lastly, the number of levels and their labels were discussed, and the parent advisory group supported the chosen labels and the removal of a middle option, with one parent highlighting that it was often difficult to choose between adjacent categories in the original PC-QoL-8.

**Table 5:** Proposed health state classification system after expert panel discussion

Item (During the past week…)	Level 1	Level 2	Level 3	Level 4
You were **awakened during the night** because of your child’s cough	Always	Often	Sometimes	Never
You felt you were being **overprotective** because of your child’s cough	Always	Often	Sometimes	Never
You felt **upset** because of your child’s cough	Always	Often	Sometimes	Never
You were worried/concerned about **leaving your child with others** because of their cough	Always	Often	Sometimes	Never
You felt **scared** because of your child’s cough	Always	Often	Sometimes	Never
You were worried/concerned about your child being able to **lead a normal life**	Always	Often	Sometimes	Never

Bold signifies main theme of the item

## Discussion

In this study, we undertook the initial necessary steps for creating the first preference-based measure for estimating spillover health utility in parents of children with chronic cough. Using a combination of classical test theory (exploratory factor analysis) and item response theory (Rasch analysis) techniques, we explored the latent factorial structure of the widely used PC-QoL-8 and proposed amendments that would make it amenable to valuation. Our analyses indicate that six dimensions, each with four levels, could be used as the health classification system for a parent-proxy child chronic cough preference-based measure. Similarly to other preference-based measures [24, 44], use of the original PC-QoL-8 in its entirety would be inappropriate for generating societal preferences, given that the 8-item version would have 7^8^ possible combinations. In a full factorial valuation study (e.g. time trade-off, standard gamble, or discrete choice experiment) there would be approximately 17 trillion pairwise combinations. The amendments we propose would have 4^6^ possible combinations and approximately 8 million full factorial combinations, which using modern, efficient blocking techniques could be valued in a study with a feasible sample size [25].

Our approach to analysis of the PC-QoL-8 differed from that of the original developers. Newcombe et al. [27, 33] who showed that the PC-QoL-8 describes 3 domains of functioning, social, physical and psychological [27]. We did not find this structure in our analysis of the short form of the PC-QoL-8; however, we did retain at least one item from each of these domains. Additionally, principal components and factor analysis indicated a correlation between all items and high loading onto one underlying factor; however, further investigation with Rasch techniques and careful examination of item wording demonstrated local independence of items, aiding in the avoidance of illogical or implausible potential health states in a valuation study the proposed classification system.

This study was limited by both the chosen sample and the methodology utilised. We used data from parents of children undertaking a diagnostic test available only in tertiary care, possibly limiting generalisability to wider populations. Secondly, half of our sample would go on to be diagnosed with bronchiectasis after further investigation following the procedure, which is thought to lie at the severe end of the protracted bacterial bronchitis > chronic suppurative lung disease > bronchiectasis spectrum [56]. This high representation of participants with more severe disease may explain observed floor effects of responses and a maximum of 8 items that could be included in our health state classification system may limit the feasibility of targeting the persons well and indeed. The wright map (ESM Fig. S9) indicates a item distribution that is narrower than that of the persons. Additionally, whilst we assessed for differential item functioning using factors more closely related to the child rather than the parent (for example, diagnosis category) due to limitations in the available data, we posit that to some extent, the chosen factors: child age, diagnosis and ethnicity do impact the parents and may be correlated with experience raising children in general, experience with chronic cough and the parent’s ethnicity, respectively. Third our sample included only patients undergoing management at tertiary facilities in two capital cities in Australia and may not be applicable to families who do not have access to these facilities or live in dissimilar societies, given the potential for vastly different experiences with health and healthcare [57]. Fourth, we used a partial credit Rasch model which has been criticised for its’ threshold disordering problem, which is not present in certain other Rasch models, for example, the method of successive dichotomisations [58]. Given our aim to reduce levels and the threshold disordering we detected, we chose to collapse levels, but where this is not the goal, use of other Rasch models can render this step unnecessary. Finally, our study was somewhat weakened by a lack of a confirmation dataset for the Rasch analysis, which has previously been recommended [26]. Despite this, we had a large sample, a panel of experts with experience working outside of major tertiary hospitals and a panel of consumers to explore the content validity of the scale, strengthening the use of this scale outside of our sample.

Despite these limitations, our analysis of the PC-QoL-8 successfully addressed the study aims. These findings represent an important step towards operationalising a preference-based measure derived for estimating spillover health utility and QALYs in parents of children with chronic wet cough pending further research to elicit societal preference weights for each health state described the PC-QoL-8-derived classification system. A preference-based measure derived from the PC-QoL-8 will allow for the direct estimation of QALYs utilising a widely used cough-specific HR-QoL measure using responses PC-QoL-8 7-level questionnaire.

### Supplementary Information

Below is the link to the electronic supplementary material.

Supplementary file1 (DOCX 680 KB)

## Data Availability

Data underlying published results, after de-identification, will be made available on reasonable request for researchers who provide a methodologically sound proposal and obtain an approval from the Human Research Ethics Committee.
