# Supplementary material for: Rasch validation of the short form (8 item) PC-QoL questionnaire and applicability of use as a health state classification system for a new preference-based measure
Source: Qual Life Res. 2024 Apr 23;33(7):1893–903. doi: 10.1007/s11136-024-03652-w (PMC11176213; doi:10.1007/s11136-024-03652-w)

Rasch validation of the short form (8 item) PC-QoL questionnaire, and applicability of use as a health state classification system for a new preference-based measure.

Jack M. Roberts^1,2^, Anne B. Chang^1,2,3^, Vikas Goyal^2,3^, Nitin Kapur^3^, Julie M. Marchant^1,2,3^, Steven M. McPhail*^1,4^, Sanjeewa Kularatna*^1,5^

Corresponding author: Jack Roberts – [jack.roberts@connect.qut.edu.au](mailto:jack.roberts@connect.qut.edu.au)

# Online resource: tables

Table S1: Descriptive statistics of PC-QoL-8 items, including mean (SD) score, Median (IQR), percentage of missing responses, and proportion responding at lowest (floor) and highest (ceiling) levels.

| Item | Mean score/ 7 | SD | Median (IQR) | Missing (%) | Floor (score = 1) % | Ceiling (score = 7) % |
| --- | --- | --- | --- | --- | --- | --- |
| awakened | 3.783742 | 2.053998 | 4 (2-5) | 0% | 19% | 18% |
| helpless | 3.855607 | 2.109476 | 4 (2-6) | 0.2% | 19% | 20% |
| lead a normal life | 4.475385 | 1.984573 | 5 (3-6) | 0.3% | 11% | 22% |
| leaving child with others | 4.322034 | 2.194865 | 4 (2-7) | 0.5% | 16% | 27% |
| not sleeping well | 3.901387 | 2.049424 | 4 (2-6) | 0.5% | 15% | 18% |
| overprotective | 4.282642 | 2.178918 | 4 (2-7) | 0.2% | 17% | 26% |
| scared | 4.349693 | 2.033615 | 4 (3-6) | 0% | 12% | 24% |
| upset | 3.978495 | 2.049277 | 4 (2-6) | 0.2% | 16% | 19% |

Table S2: Measures of Sampling Adequacy (MSA)

| Item | Item MSA |
| --- | --- |
| upset | 0.9154166 |
| helpless | 0.9349885 |
| scared | 0.9039992 |
| overprotective | 0.9248963 |
| awakened | 0.8638436 |
| not sleeping well | 0.8957670 |
| lead a normal life | 0.9470386 |
| leaving child with others | 0.9116123 |
| Mean MSA: 0.912 | |

Table S3: Confirmatory factor analysis model fit statistics

| Model | Χ^2^ | Degrees of freedom | p value | CFI | RMSEA | SRMR |
| --- | --- | --- | --- | --- | --- | --- |
| 3 Factor | 143.901 | 17 | 0.000 | 0.958 | 0.108 | 0.038 |

Table S4: Eigenvalues of factors extracted by principal components analysis indicate retention of only one factor for further investigation (eigenvalue > 1).

| Factor | Eigenvalues |
| --- | --- |
| 1 | 4.9443855 |
| 2 | 0.7975295 |
| 3 | 0.5667637 |
| 4 | 0.4298173 |
| 5 | 0.3717218 |
| 6 | 0.3263879 |
| 7 | 0.2959447 |
| 8 | 0.2674496 |

Table S5: Results of Rasch analysis after recoding item levels, before removing any items

| Item (“During the past week how often…”) | | Statistics after item level collapsed | | | |
| --- | --- | --- | --- | --- | --- |
|  |  | Residual | χ^2^ | P-value | DIF |
| 1. Did you feel **helpless** because of your child’s cough? | | -0.278 | 7.679 | 0.262567 | Yes (ethnicity, diagnosis) |
| 2. Were you worried/ concerned about your child **not sleeping** because of cough? | | 0.29 | 4.742 | 0.577318 | No |
| 3. Did you feel **overprotective** because of your child’s cough? | | 0.739 | 8.416 | 0.209162 | No |
| 4. Did you feel **upset** because of your child’s cough? | | -1.686 | 14.946 | 0.020683 | No |
| 5. Were you worried/ concerned about **leaving your child with others** because of their cough? | | 0.085 | 6.205 | 0.400589 | No |
| 6. Did you feel **scared** because of your child’s cough? | | -0.827 | 13.055 | 0.042176 | No |
| 7. Were you worried/ concerned about your **child being able to lead a normal life**? | | 2.343 | 5.645 | 0.464052 | Yes (age) |
| 8. Were you **awakened during the night** because of your child’s cough? | | 2.518^a^ | 6.085 | 0.413693 | No |
| Overall model statistics: | Total - item trait χ^2^ = 66.774, p = 0.0377^b^  Person separation index = 0.85  Person location: mean (SD) = 0.144 (1.595)  Person fit residual: mean (SD) = -0.445 (1.481) ^c^  Item location: mean (SD) = 0.00 (0.240)  Item fit residual: mean (SD) = 0.398 (1.454) ^c^  Person fit standard error: Mean = 0.330586 | | | | |
| ^a^ Extreme fit residual value >±2.5 threshold, ^b^ Bonferroni adjusted alpha: 0.05/8 = 0.00625, ^c^ fit residual SD threshold = 1.5 | | | | | |

Table S6: Residual correlation matrix of PC-QoL after removal of item 1 and item 7

|  | Item 2 | Item 3 | Item 4 | Item 5 | Item 6 | Item 8 |
| --- | --- | --- | --- | --- | --- | --- |
| Item 2 | 1 |  |  |  |  |  |
| Item 3 | -0.228 | 1 |  |  |  |  |
| Item 4 | -0.295 | -0.215 | 1 |  |  |  |
| Item 5 | -0.227 | -0.046 | -0.226 | 1 |  |  |
| Item 6 | **-0.337*** | -0.205 | 0.076 | -0.134 | 1 |  |
| Item 8 | 0.131 | **-0.335*** | -0.205 | **-0.352*** | **-0.303*** | 1 |
| *Magnitude of residual correlation >0.3 | | | | | | |

# Online resource: figures


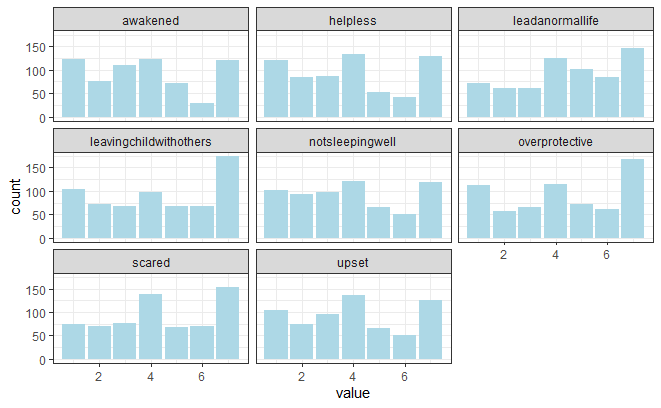


Figure S1: Histograms of response frequency to PC-QoL 8 items. No item was normally distributed, and most had high floor or ceiling effects.


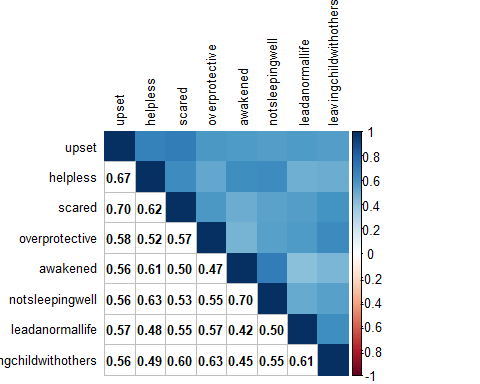


Figure S2: Correlation plot/ matrix of PC-QoL 8 items


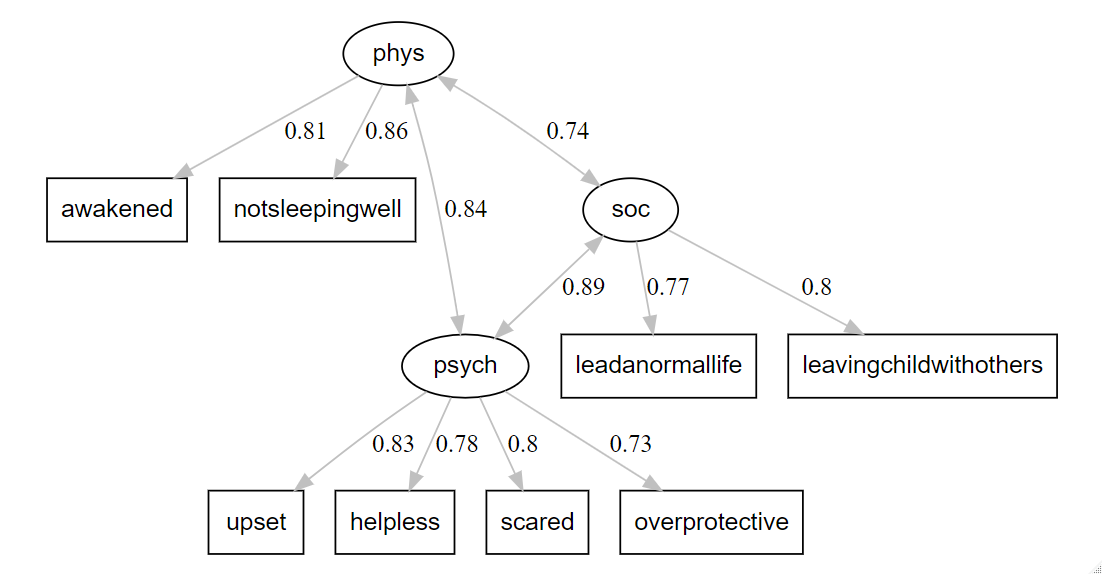


Figure S3: Path diagram of 3 factor (Newcombe) confirmatory factor analysis.


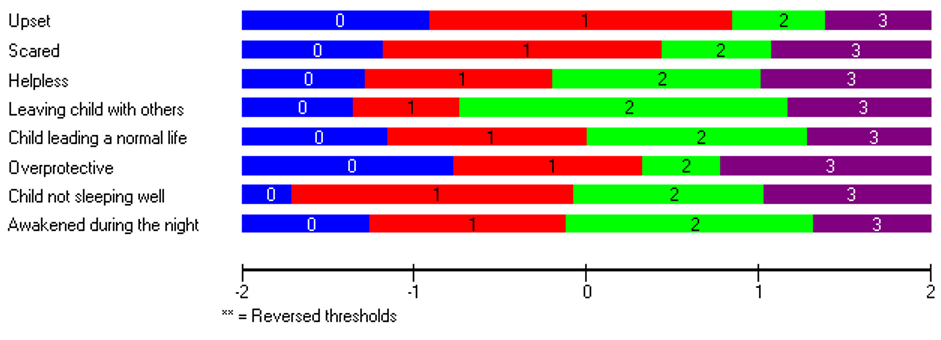


Figure S4: Category probability curves of each item on the unmodified scale. Each item demonstrates threshold disordering.


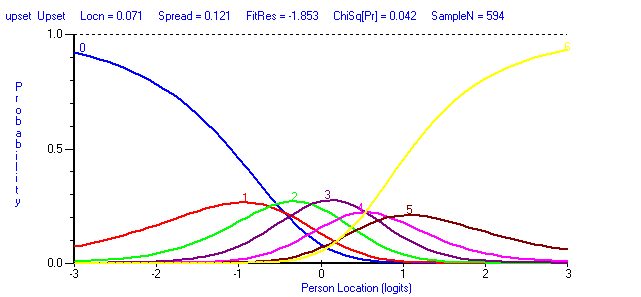

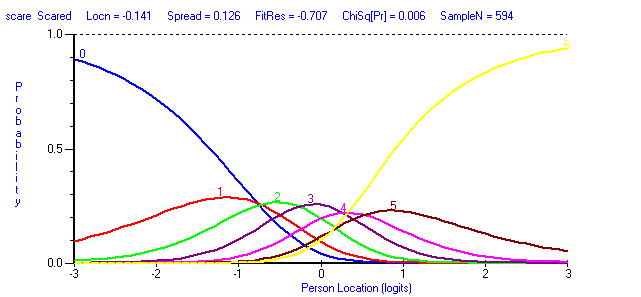

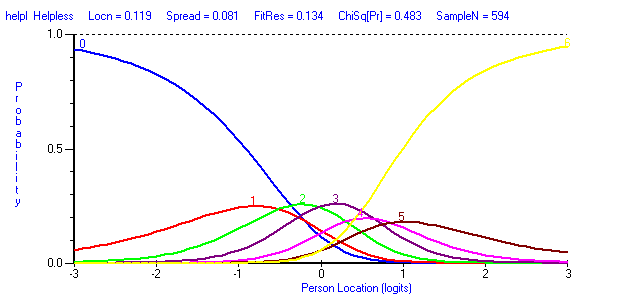

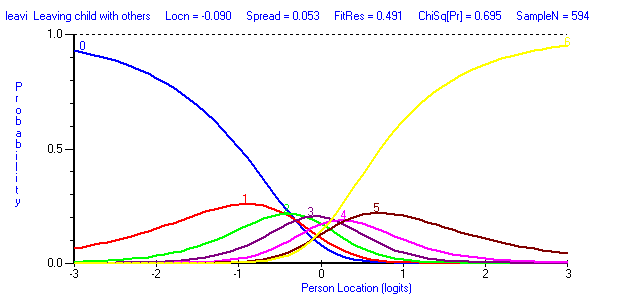

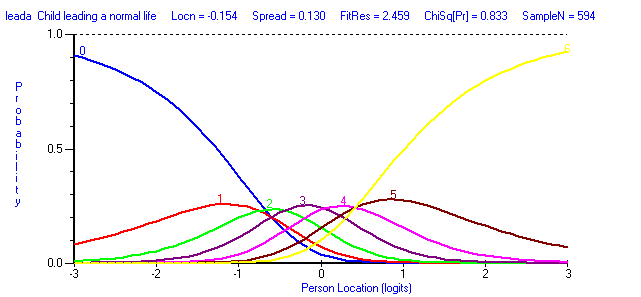

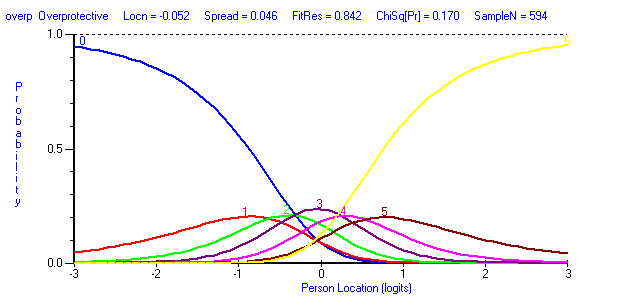

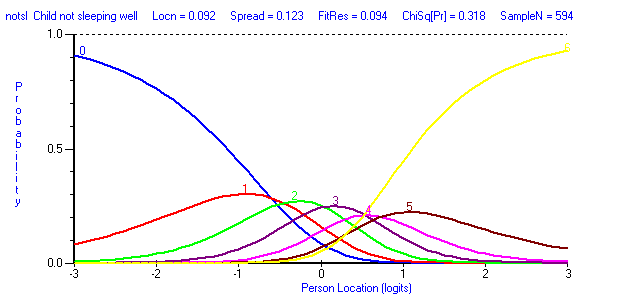

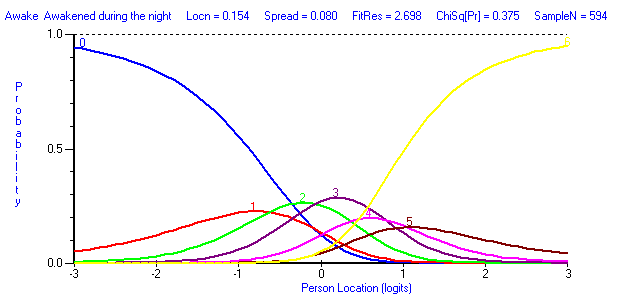


Figure S5: Item threshold map after collapsing item levels, before removing any items


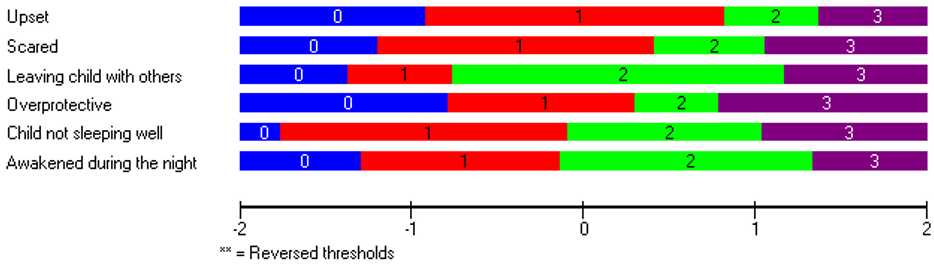


Figure 6: Category probability curves of each item on the scale after modification. Category thresholds are now ordered after level reduction.


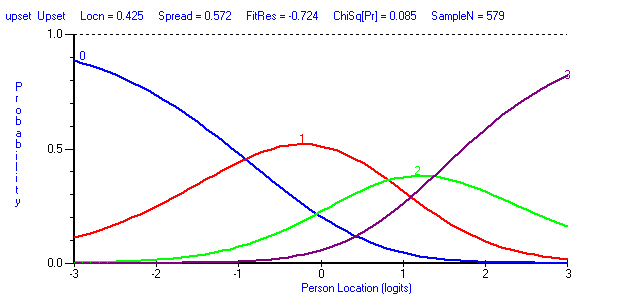

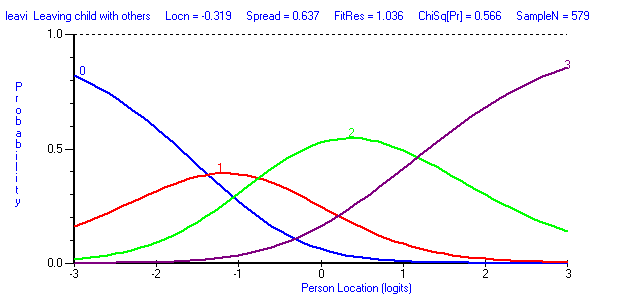

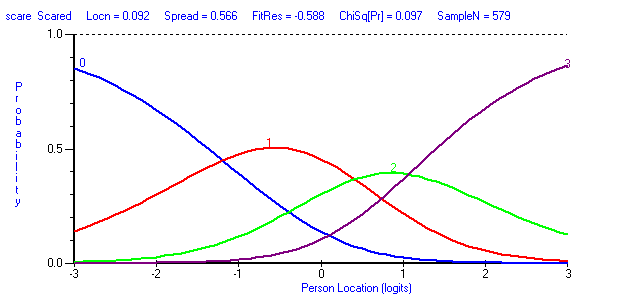

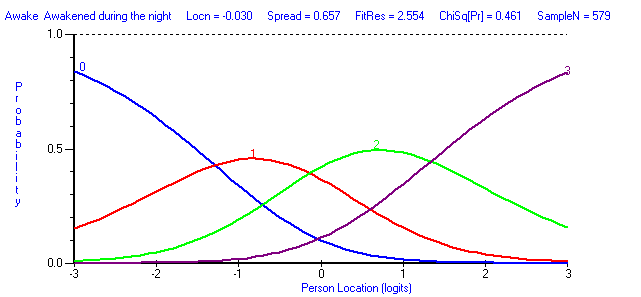

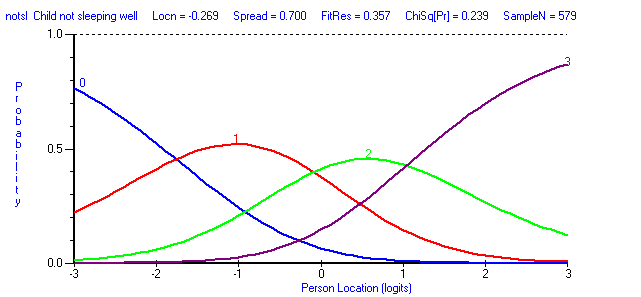

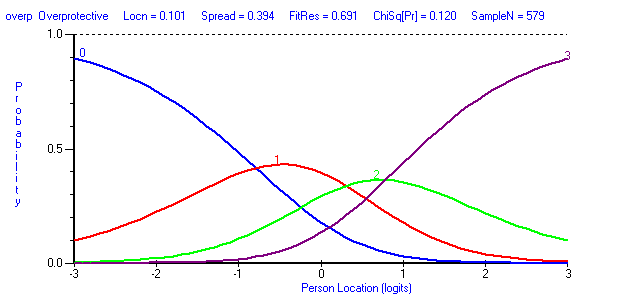


Figure S7: Item threshold map of PC-QoL after removal of item 1 & item 8 due to differential item functioning

Figure S8: Person-item threshold distribution graph of PC-QoL after removal of item 1 & item 8 due to differential item functioning


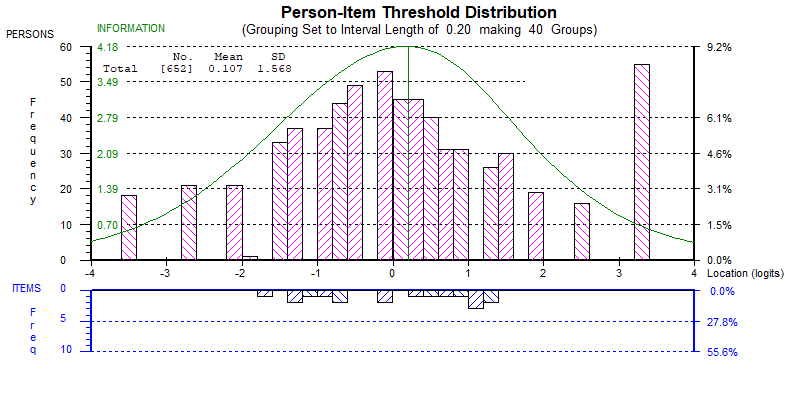


Figure S9: Wright map of PC-QoL after removal of item 1 & item 8 due to differential item functioning


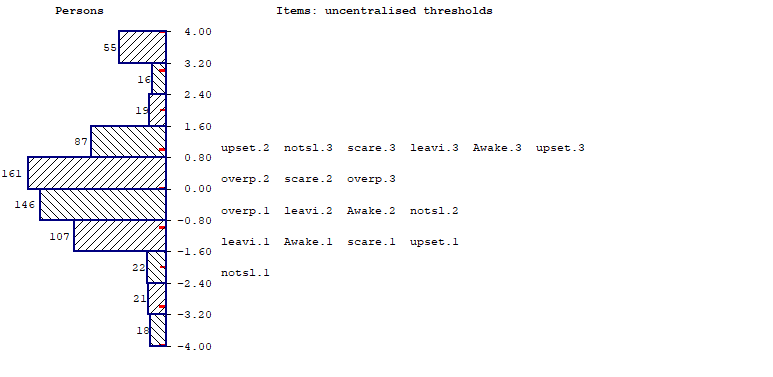

Supplement: Supplementary file 1 — Supplementary file1 (DOCX 680 KB) [file 11136_2024_3652_MOESM1_ESM.docx]
